# DUX4 Signalling in the Pathogenesis of Facioscapulohumeral Muscular Dystrophy

**DOI:** 10.3390/ijms21030729

**Published:** 2020-01-22

**Authors:** Kenji Rowel Q. Lim, Quynh Nguyen, Toshifumi Yokota

**Affiliations:** 1 Department of Medical Genetics, Faculty of Medicine and Dentistry, University of Alberta, Edmonton, AB T6G2H7, Canada; kenjirow@ualberta.ca (K.R.Q.L.); nguyenth@ualberta.ca (Q.N.); 2 The Friends of Garrett Cumming Research & Muscular Dystrophy Canada, HM Toupin Neurological Science Research Chair, Edmonton, AB T6G2H7, Canada

**Keywords:** facioscapulohumeral muscular dystrophy, double homeobox protein 4 (DUX4), skeletal muscle, toxicity, cell death, muscle differentiation, embryonic gene expression, signalling, epigenetics

## Abstract

Facioscapulohumeral muscular dystrophy (FSHD) is a disabling inherited muscular disorder characterized by asymmetric, progressive muscle weakness and degeneration. Patients display widely variable disease onset and severity, and sometimes present with extra-muscular symptoms. There is a consensus that FSHD is caused by the aberrant production of the double homeobox protein 4 (DUX4) transcription factor in skeletal muscle. DUX4 is normally expressed during early embryonic development, and is then effectively silenced in all tissues except the testis and thymus. Its reactivation in skeletal muscle disrupts numerous signalling pathways that mostly converge on cell death. Here, we review studies on DUX4-affected pathways in skeletal muscle and provide insights into how understanding these could help explain the unique pathogenesis of FSHD.

## 1. Introduction: Facioscapulohumeral Muscular Dystrophy and DUX4

Facioscapulohumeral muscular dystrophy (FSHD) is an autosomal dominant disorder primarily characterized by asymmetric, progressive muscle weakness. Muscles of the face, shoulders, and upper limbs are typically affected first, followed by those of the lower extremities and other muscles [1]. Symptoms usually manifest during the second or third decade of life, but cases have been reported to present anywhere from birth to adulthood [2,3]. In particular, a rare, early-onset form of FSHD that occurs before 10 years of age shows increased severity compared to the rest of FSHD patients [3,4]. On that note, disease severity is highly variable among individuals, with 20% of FSHD-mutation carriers remaining asymptomatic [5,6]. While life expectancy is not affected in FSHD, it can be a disabling disorder. Wheelchair use is required by 20% of patients, with patients also having an increased risk of fatigue and chronic pain [1,7]. About 1 in 8000 to 22,000 individuals are affected with FSHD worldwide, making it the third most common inherited form of muscular dystrophy [1,8].

Two different but related genetic mechanisms cause FSHD, classifying patients as having either FSHD1 or FSHD2. FSHD1, affecting 95% of patients, is caused by contractions (i.e., reductions in repeat unit content) of a macrosatellite repeat array found at the distal end of chromosome region 4q35 [9,10]. This array normally consists of 11–100 3.3-kb D4Z4 repeat units, each containing two exons and the entire open reading frame (ORF) of the double homeobox protein 4 (*DUX4*) gene [11]. The array is also normally hypermethylated in healthy individuals [12]. Contraction of the array to 10 or fewer D4Z4 units with the presence of a permissive 4qA haplotype was generally thought to disrupt its methylation, leading to chromatin relaxation at the array and allowing for *DUX4* transcription [13,14,15]. The third exon of *DUX4* is found directly after the last D4Z4 unit in the array. In the 4qA haplotype, this exon contains a polyadenylation signal that ensures production of the *DUX4* transcript [14]. On the other hand, in FSHD2, representing 5% of FSHD patients, contractions are also observed in the D4Z4 array but not to the same extent as in FSHD1 (an average 12–16 D4Z4 units is reported) [16,17]. The more causative mutations for FSHD2 are those in genes coding for proteins involved in D4Z4 array methylation, e.g., *SMCHD1* and *DNMT3B* [18,19]. With the 4qA haplotype, a similar loss of epigenetic silencing of the D4Z4 array is observed with these mutations, resulting in *DUX4* transcription. While strict cut-offs such as ≤10 D4Z4 units were previously used to define FSHD types, it is now known that this is not always the case. Asymptomatic mutation carriers with 7–10 D4Z4 units have been described [20]. FSHD2 patients with ≤10 D4Z4 units have also been found [21]. These suggest a more complex interplay of genetic and epigenetic factors in the manifestation of the disease than initially thought, inviting a re-evaluation of the distinction between FSHD1 and FSHD2.

Given its presence in the D4Z4 array, extensive investigations have been made into the role of *DUX4* in FSHD pathogenesis. The *DUX4* transcript is alternatively spliced into various isoforms, some of which are expressed in non-muscle tissues such as the testis or thymus [17,22,23]. Here, we focus on the full-length isoform expressed in skeletal muscles in FSHD, which consists of the first three *DUX4* exons on the 4qA haplotype. We refer to this isoform as *DUX4* from this point on. *DUX4* codes for a transcription factor with two homeobox domains near the N-terminus and a transcriptional activation domain by the C-terminus; the protein mostly functions as an activator of gene expression [24]. Using RNA-sequencing on extracts derived from *DUX4*-transduced myoblasts, patient-derived primary muscle cells, and patient biopsies, one group consistently observed that the majority of significantly affected genes in FSHD were DUX4 transcriptional targets [25]. Together with findings from similar studies and lines of evidence demonstrating the cytotoxicity of DUX4, there now appears to be a consensus that FSHD is caused by aberrant *DUX4* expression in muscle [1,17]. However, the mechanisms explaining how DUX4 can lead to FSHD pathology remain an area of active research.

*DUX4* is normally expressed during the 4-cell stage in human embryos to initiate zygotic gene activation [26,27] and is epigenetically silenced thereafter for the rest of development and life, only retaining expression of certain isoforms in the testis and thymus. A corresponding finding was found for the murine *DUX4* homolog *Dux* in 2-cell stage embryos [26,27]. The reactivation of *DUX4* expression in FSHD patients therefore presents a peculiar problem, that of determining what effects an “embryonic” gene could have in a mostly differentiated environment. In this review, we provide a summary of knowledge the field has gained so far on DUX4 and its place in skeletal muscle signalling and development, as well as how its activities contribute to FSHD pathogenesis. We begin by describing the clinical features of FSHD, to better contextualize the molecular and cellular changes that will be discussed.

## 2. Clinical Characteristics of FSHD

### 2.1. Skeletal Muscle Manifestations

Despite their genetic differences, FSHD1 and FSHD2 are phenotypically indistinguishable [16,28]. The classic form of FSHD is characterized by muscle weakness and wasting involving mostly the facial, scapular and upper arm muscles [29]. FSHD has a distinctive pattern of skeletal muscle weakness and a wide spectrum of disease severity. The age of onset varies from infancy to middle age, but the majority of patients develop signs and symptoms in their late teens to the early 20s [1]. Muscle weakness and atrophy start in the face and shoulder muscles, progressing to the upper arms, trunk muscles and lower extremities, typically evident first in the anterior leg muscles followed by the thigh and pelvic girdle muscles [28]. Unlike most other dystrophies, asymmetric involvement is typical and more pronounced in FSHD, and contractures are absent or minimal.

Weakness involving the facial muscles, especially the orbicularis oculi and orbicularis oris (eyelid and lip muscles, respectively), is usually the initial symptom of FSHD [1,30]. Orbicularis oculi weakness can manifest as sleeping with eyes slightly open, which can lead to dry eyes and other ocular problems. Weakness in the orbicularis oris often presents as a mild dimpling in the areas lateral to the angles of the mouth, an inability to whistle, difficulty drinking through a straw, difficulty in puckering of the lips, or everted lips in severe cases. Facial weakness can be absent or mild early in the course of disease and can remain mild for many years later [29,31].

Other common initial symptoms of FSHD are related to shoulder and upper arm muscle weakness [30,32]. Patients with periscapular muscle weakness often find it difficult to raise their arms over the head or lift objects above shoulder level. Weak shoulder muscles tend to make the scapula protrude from the back, a common sign known as scapular winging. However, this is typically a unilateral finding owing to asymmetric muscle involvement. Due to selective involvement of the lower versus the upper trapezius, attempts to forward flex or abduct the shoulders result in the distinctive upward jutting of the scapula [1,29,30]. The deltoid muscles are relatively spared in the early stages and are relatively less affected compared to the other shoulder girdle muscles. The forearm muscles are usually spared, however, the biceps and triceps often experience weakness and atrophy as the disease progresses. On the contrary, the pectoral muscles are severely affected in FSHD, which becomes evident in FSHD patients with the appearance of prominent axillary creases [30,32].

As the disease progresses, the muscles of the trunk and lower extremities also become affected [28]. Weakness of the abdominal muscles results in a protuberant abdomen in patients. Selective involvement of the lower abdominal muscle results in an upward deflection of the umbilicus upon attempted neck flexion in the supine position, or a positive Beevor’s sign [33]. Progressive paraspinal muscle involvement manifests as an exaggerated form of lumbar lordosis. Muscle weakness in the hips and pelvis makes it difficult for FSHD patients to climb stairs or walk long distances [1,30]. Weakness in the lower leg muscles may also lead to a condition named foot drop, which affects mobility and increases the risk of falls [29,32].

Histological analysis reveals that skeletal muscles from FSHD patients display widely variable morphology [1]. This may be due to the asymmetric clinical involvement of muscles in the disease, limitations concerning the location of biopsy collection, or both. Approximately 10-15% of FSHD biopsies appear to be histologically normal, with the rest showing anywhere from minimal to severe morphological changes [1]. Observed histopathological characteristics of FSHD include increased fiber size variability, the presence of degenerating and regenerating fibers, fibrofatty replacement, fibrosis, and inflammation—the extent of which varies among patients. In particular, inflammation is observed in about a third of patient skeletal muscle biopsies, and is not only endomysial but also distinctly perivascular compared to other muscular dystrophies [1,34]. FSHD1 and FSHD2 muscle biopsies show similar histopathology, with no significant differences in pathology grade as determined by a trained neuromuscular pathologist in one study using a 12-point scoring system of histological characteristics [34].

### 2.2. Extramuscular Manifestations

Respiratory involvement is infrequent in patients with FSHD. A cross-sectional observational study of 69 FSHD patients reported restrictive lung disease on pulmonary function testing in 10% of patients [35]. Restrictive lung disease is more common in patients with severe disease manifestations, especially those with significant trunk and hip girdle weakness or those who are wheelchair-bound [35,36]. A Dutch population study estimated that ~1% of patients with FSHD require ventilatory support [36]. Cardiac function is typically preserved in FSHD, unlike many other muscular dystrophies. Mild cardiac conduction abnormalities, in particular supraventricular arrhythmias, were reported in ~5% of patients [37]. Asymptomatic right bundle branch block was also reported to have a higher prevalence in FSHD compared to the general population [38]. 

High-frequency hearing loss and retinal vascular disease are present in some FSHD patients [39,40]. Hearing loss in children with FSHD seems to correlate with larger D4Z4 contractions [41,42]. The retinal vasculopathy phenotype is characterized by bilateral retinal telangiectasia and microaneurysms that can rarely result in exudative retinopathy, a condition known as Coats disease [42]. It is estimated that ~0.6% of FSHD patients develop Coats disease [42].

### 2.3. Early-Onset FSHD

The early-onset or infantile form of FSHD is defined by facial weakness before the age of 5 and scapular weakness before the age of 10 [43]. It is estimated to occur in approximately 4%–21% of FSHD patients [4]. The patterns of muscle weakness observed in early-onset FSHD, as well as its systemic features, are similar to those found in classic FSHD. However, the infantile form differs in that it is characterized by increased severity, faster disease progression, and more frequently occurring systemic features [4,44,45]. Furthermore, some systemic features such as cognitive disability or epilepsy are only associated with early-onset FSHD [43].

A systematic study of 227 patients with early-onset FSHD illustrated the severity of muscle weakness in this population [44]. The percentage of wheelchair-dependent patients was significantly higher in the early-onset group (40%) compared to the general FSHD population (6.4%). Spinal deformities and the use of assisted ventilation were highly prevalent in early-onset FSHD. In particular, the most frequent systemic feature observed was sensorineural hearing loss of high-pitched sounds, which manifested in 40% of early-onset FSHD patients. These patients also exhibit a higher incidence of clinical vision loss than the classic FSHD population.

## 3. DUX4 in Skeletal Muscle Signalling, Growth, and Development

In 1999, Gabriëls et al. discovered that the D4Z4 repeat unit contained the sequence for a putative protein that contained two homeobox domains, which the authors called DUX4 [11]. They further determined by in vitro reporter assays that part of the sequence preceding the *DUX4* ORF in the D4Z4 repeat had promoter activity. Endogenous *DUX4* expression is extremely low, however (we now know only 1/1000 myoblast or 1/200 myotube nuclei in patient primary cells are DUX4-positive by immunofluorescence [46]). This led to difficulties in detecting *DUX4* expression from patient samples, preventing inquiry into whether or not DUX4 was a key player in FSHD pathogenesis. Improvements in the knowledge of the *DUX4* gene, technique, and reagent availability eventually confirmed the presence of *DUX4* mRNA and protein in FSHD primary muscle cells [47] nearly a decade later, strengthening the link between muscle-specific *DUX4* expression and FSHD.

**Figure 1:**
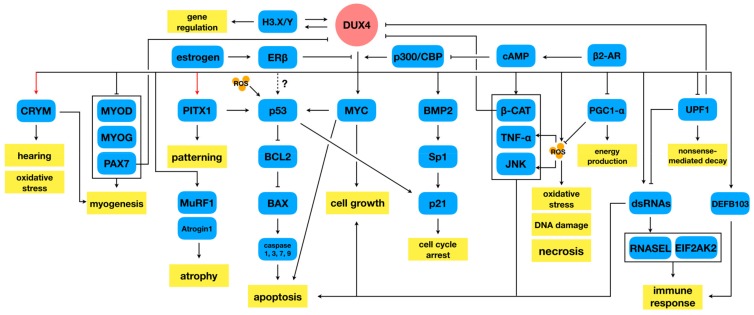
DUX4 signalling in FSHD-affected skeletal muscle. A simplified overview of the various signalling activities of DUX4 discussed in this review is depicted. Red arrows indicate a confirmed direct downstream DUX4 transcriptional target. Abbreviation: ROS, reactive oxygen species; FSHD facioscapulohumeral muscular dystrophy; DUX4: double homeobox protein 4.

DUX4 has since been implicated as being involved in cell death, oxidative stress, muscle differentiation and growth, epigenetic regulation, and a number of other signalling pathways in skeletal muscle. While most of these investigations were launched to try and explain the mechanism behind DUX4-mediated cytotoxicity, they have also been instrumental in helping us understand the basic biology of FSHD. Figure 1 shows a simplified overview of the various DUX4 signalling pathways that will be discussed in this review.

### 3.1. Cell Death

The degeneration of skeletal muscle in FSHD suggests that DUX4 may be initiating cell death pathways. Indeed, Kowaljow et al. (2007) found that overexpression of *DUX4 in vitro* resulted in significant cell death that was accompanied by significant increases in released lactate dehydrogenase into the medium, emerin redistribution, and caspase 3/7 activity [48]. Flow cytometry revealed an increased proportion of annexin V-positive cells when *DUX4* was transfected. Altogether, these findings point out a possible role for DUX4 in apoptosis.

Wallace et al. (2011) injected wild-type mice intramuscularly with adeno-associated viral (AAV) vectors containing a *DUX4* construct and collected samples for testing on a quantitative real-time PCR array for apoptosis-associated genes [49]. A third of the significantly up-regulated genes were involved in the p53 pathway, which is primarily known for regulating intrinsic or mitochondrial apoptosis [50,51]. Chemical inhibition of p53 pathway members (p53, caspase-1, and Bax) significantly decreased DUX4-mediated caspase-3/7 activation in vitro in *DUX4*-transfected HEK293 cells, a finding corroborated by a later study using different inhibitors [52]. Finally, *p53* knockout mice injected intramuscularly with AAV-*DUX4* had transduced muscles that were histologically normal [49], suggesting that *DUX4*-induced cell death depends on the p53 pathway.

This dependence of *DUX4* toxicity on p53 is contested, however. To further study this relationship, Bosnakovski et al. (2017) used a modified version of the immortalized mouse C2C12 myoblast line that contains a doxycycline-inducible *DUX4* transgene (iC2C12-DUX4) [53]. Surprisingly, transfection of doxycycline-treated iC2C12-DUX4 cells with constructs of known p53 inhibitors such as a dominant-negative p53 mutant, MDM2, and TRIM24 all did not significantly affect the level of death observed in these cells. The authors confirmed their results by crossing iDUX4(2.7) mice onto a *p53* knockout background. Despite carrying a doxycycline-inducible *DUX4* transgene, iDUX4(2.7) mice still express basal levels of *DUX4*, which lead to embryonic lethality or 100% mortality in males by 6 weeks of age. Loss of *p53* did not prevent this lethal phenotype, as no *DUX4*-expressing males were obtained from the cross. Results from other studies [54,55] and analysis of previous microarray and RNA-sequencing data cast further doubt on the dependence of DUX4-mediated toxicity on the p53 pathway. As suggested by the authors, the high levels of *DUX4* expression or the viral method of *DUX4* introduction used in the previous study [49] could have led to the observed involvement of the p53 pathway. Since the previous studies also used chemical inhibition to implicate p53 and its targets in DUX4-mediated toxicity, investigating the specificity of these inhibitors may help explain this disparity.

The relationships of DUX4 with other genes and proteins involved in apoptosis were also investigated. One such gene is *CDKN1A*, which codes for the cyclin-dependent kinase inhibitor p21. p21 arrests the cell cycle in the presence of stressful stimuli, such as those that may initiate the intrinsic apoptosis pathway [56]. Interestingly, *CDKN1A* is one of the downstream targets of the p53 transcription factor, and p53-dependent cell cycle arrest is thought to be mostly through p21. *DUX4* overexpression *in vitro* increases *CDKN1A* expression, which in the two studies that observed this, was not accompanied by *p53* upregulation [55,57]. The effect was dependent on the integrity of Sp1 transcription factor binding sites on the *CDKN1A* promoter [57]. *DUX4* may be activating *CDKN1A* expression through the Bmp2 signalling pathway, which acts upstream of Sp1. Knockdown of *CDKN1A* improved the proliferative capacity of *DUX4*-transfected cells *in vitro*, indicating its potential contribution to the toxicity of DUX4.

Another apoptosis-related gene that DUX4 upregulates is *MYC*. Shadle et al. (2017) generated a rhabdomyosarcoma cell line stably transduced with doxycycline-inducible *DUX4* (RD-DUX4i), which they proceeded to transfect with a siRNA library to search for genes that enable DUX4-mediated toxicity [54]. Among the genes found to improve RD-DUX4i viability was *MYC*, a well-known proto-oncogene whose protein product MYC (or c-Myc) functions in cell cycle progression and in universal transcriptional activation [58,59]. Furthermore, MYC can trigger both extrinsic and intrinsic pathways of apoptosis. *DUX4* overexpression increases MYC protein levels indirectly by increasing *MYC* mRNA stability (we will review *DUX4* effects on transcript quality control later), ultimately increasing activation of MYC downstream targets [54]. Increased *MYC* transcript levels were confirmed in FSHD muscle cells. The same study found that *DUX4* expression increased nuclear double-stranded RNA (dsRNA) accumulation, which can initiate a signalling cascade that globally inhibits translation and leads to apoptosis.

To obtain a more integrative model of the signalling pathways affected in FSHD, Banerji et al. (2015) used a meta-analysis approach in tandem with a differential network algorithm to identify an “FSHD-specific disease network” [60]. This study identified β-catenin as a central point in the disease network, suggesting that the perturbation of its interactions mostly distinguishes the FSHD phenotype. β-catenin is a vital member of the canonical Wnt signalling pathway, which has roles in muscle differentiation and repair, as well as embryonic development [61,62]. Analysis of the network suggested that Wnt/β-catenin signalling was activated in FSHD. This was confirmed *in vitro*, with *DUX4*-transfected C2C12 myoblasts having increased Wnt/β-catenin signalling. Moreover, the network identified crosstalk among the Wnt/β-catenin, TNF-α, and JNK signalling pathways. The latter two are involved in oxidative stress-induced cell death, thereby positioning β-catenin as a major contributor to DUX4-mediated toxicity.

Due to the involvement of Wnt/β-catenin signalling in retinal vasculature development and hearing (via JNK) this pathway has been thought to explain the connection between the muscular and extra-muscular manifestations seen in FSHD [60]. An earlier study discovered that the same pathway is involved in the transcriptional repression of *DUX4* [52]. This consequently results in a negative feedback loop wherein *DUX4* activates Wnt/β-catenin signalling, which represses its own expression. This may explain why only a few DUX4-positive nuclei are found in FSHD-affected skeletal muscle cells at any one time [46]. Should this be true, it would be interesting to investigate the mechanisms that allow for this escape from Wnt/β-catenin-mediated repression, and how this in turn leads to the chronic over-activation of Wnt/β-catenin signalling in FSHD.

### 3.2. Oxidative Stress

Oxidative stress-induced damage is increasingly being recognized as a hallmark of FSHD [63]. FSHD skeletal muscle biopsies contain evidence of oxidative stress and damage, e.g., protein carbonylation and accumulation of lipofuscin [64]. Additionally, both muscle biopsies and blood samples from patients have increased levels of antioxidants compared to healthy controls [64]. High amounts of reactive oxygen species (ROS) in cells not only damage DNA, proteins, and lipids but also initiate apoptotic and necrotic pathways of cell death [65]. Indeed, DNA damage has been observed in FSHD patient-derived myoblasts, and impairs their differentiation into myotubes [66].

*DUX4* expression in iC2C12-DUX4 myoblasts has been shown by Bosnakovski et al. (2008) to make these cells more susceptible to oxidative stress-induced death [55]. This corresponds with results from a previous study that reported a similarly increased susceptibility to oxidative stress in FSHD myoblasts [67]. Antioxidant use improved viability, but the rescue was incomplete and did not improve morphology [55]. A few years later, the same group performed a screen for drug-like compounds to search for potential inhibitors of DUX4 toxicity [68]. Of the compounds that tested positive, 60% had an antioxidative function, highlighting the importance of the oxidative stress pathway in DUX4 toxicity. In a different study, antioxidants ameliorated DNA damage in FSHD patient-derived myoblasts and in this case significantly improved their morphology upon differentiation [66].

While dysregulation of genes involved in oxidative stress pathways have been shown in multiple studies [55,60,69], only a few have started to tease out the relationships between these genes and DUX4. We mentioned how Banerji et al. (2014) showed that DUX4 activates the TNF-α and JNK signalling pathways, increasing susceptibility to oxidative stress-induced death [60]. Using the iC2C12-DUX4 model, induction of *DUX4* expression was found to repress the transcription of glutathione redox genes [55]. This increases ROS levels, activating TNF-α, which proceeds to activate JNK and initiate cell death. TNF-α also activates pathways that produce the antioxidant MnSOD via NF-κB [70,71]. Despite this, MnSOD levels are reduced in FSHD indicating that other factors may be suppressing NF-κB signalling [60,64]. Other proteins that DUX4 has been discovered to affect in the context of oxidative stress are HIF1-α (increasing sensitivity to oxidative stress) [60], ATM (increasing *DUX4* expression in response to oxidative stress) [72], and PGC1-α (activating the oxidative stress response; suppressed by DUX4) [73]. PGC-1α, in particular, is involved in pathways promoting angiogenesis via VEGF, which could explain the vascular abnormalities observed in FSHD [73,74]. Finally, FSHD myoblasts appear to be capable of handling oxidative stress up to a certain level, beyond which they become vulnerable to its effects [75]. Variation in the ability of individual muscles to handle oxidative stress may partially explain the asymmetric phenotype of muscle weakness observed in FSHD.

### 3.3. Muscle Development

*DUX4* expression has been demonstrated to downregulate genes involved in myogenesis, such as those coding for MyoD, myogenin, desmin, and Pax7; *Myf5* expression levels, on the other hand, were increased by *DUX4* [55]. Low levels of *DUX4* expression decreased muscle differentiation in vitro, as confirmed by a reduction in myosin heavy chain (MyHC)-positive fibers [55,76]. This DUX4-induced suppression of myogenic genes is found in both murine and human in vitro models [55,76,77]. Furthermore, DUX4 decreased myogenic gene expression in satellite cells, which not only reduced their proliferation but also impaired the differentiation and fusion of myotubes derived from them [77]. Transcriptomic analysis revealed that DUX4 created an overall less-differentiated state of gene expression in myoblasts [77], agreeing with the above observations.

The relationship between DUX4 and Pax7 is perhaps one of the best studied in the field. Both proteins contain homeobox domains, which exhibit significant amino acid sequence similarity with one another, e.g., DUX4 and Pax7 homeodomains show 100% identity in their DNA-binding amino acids [55,78]. Bosnakovski et al. (2017) replaced either one or both DUX4 homeodomains with those of Pax7, and found that all constructs still led to significant cell death upon transfection *in vitro* in myoblasts, indicating the functionally interchangeable nature of these homeodomains [78]. In the same study, they showed that only Pax7 or its homolog Pax3 could phenotypically compete with DUX4, among a set of proteins that also possessed homeodomains similar to DUX4. An earlier study by the same group found that *Pax7* or *Pax3* overexpression could inhibit DUX4-mediated toxicity in a dose-dependent manner, as well as significantly restore myogenic gene expression [55]. Transcriptomic studies identify Pax7 target gene repression to be a prominent feature of FSHD skeletal muscles, and may be a superior biomarker than the DUX4 target gene signature in terms of discriminating FSHD-affected muscle cells from healthy ones [79,80]. Interestingly, one study showed that DUX4 and Pax7 were never observed in the same nuclei at the same time in muscle fibers differentiated from induced pluripotent stem cells [81]. Additional layers of regulation may be involved in the competitive relationship between DUX4 and Pax7, at least in this model.

Estrogen also seems to play a role in modifying the toxic effects of *DUX4* on muscle differentiation. This follows from clinical studies reporting that female FSHD patients are less severely affected than males, and that there are more female than male asymptomatic FSHD-mutation carriers [6]. Teveroni et al. (2017) showed that 17β-estradiol (E_2_) treatment could improve the differentiation of FSHD myoblasts in vitro, as well as of myoblasts transfected with *DUX4* [82]. The authors found that E_2_ mediates this effect through ERβ and not ERα, and that E_2_ interfered with DUX4 transcriptional activity by preventing it from binding its target promoters. *DUX4* expression was not affected by E_2_, but E_2_ did redistribute DUX4 more to the cytoplasm in differentiated muscle cells.

Other genes implicated in myogenesis are affected by DUX4. In addition to the above, DUX4 activates the expression of the muscle-specific E3 ubiquitin ligases Atrogin1 (or MAFbx) and MuRF1, which are involved in protein degradation and muscle atrophy [83]. β2-adrenergic receptor (β2-AR) signalling has been associated with regulating the expression of these two genes. Treatment of FSHD patient-derived muscle cells with β2 agonists considerably inhibited *DUX4* expression and antagonized its effects [84]—Atrogin1 and MuRF1 may likely be involved in mediating the amelioration observed here. DUX4 also directly binds the promoter of *CRYM*, upregulating its expression and increasing the levels of its protein product *in vitro* [83]. CRYM (or μ-crystallin) is a reduced nicotinamide adenine dinucleotide phosphate (NADPH)-dependent thyroid-hormone binding protein that regulates the metabolic plasticity and contractility of skeletal muscles [85]. *CRYM* is also expressed in the cochlea and vestibule of the inner ear. Mutations in *CRYM* have been found to cause hearing loss [86], potentially explaining the occurrence of this phenotype in some FSHD patients. Finally, DUX4 induces the expression of the *RET* receptor tyrosine kinase (RTK) gene, which promotes the proliferation of satellite cell-derived myoblasts and maintains them in an undifferentiated state [87]. Treatment with sunitinib, an RTK inhibitor, inhibited Ret signalling and rescued differentiation in both mouse myoblasts expressing *DUX4* and FSHD patient-derived myoblasts.

### 3.4. Transcript Quality Control

We have described how DUX4 can initiate the accumulation of dsRNA foci in the nuclei of muscle cells and how this can induce apoptosis [54]. These dsRNAs are mostly derived from repetitive sequences, e.g., *Alu* and LINE-1 [88]. Interestingly, dsRNAs based on HSATII repeats were induced specifically by DUX4 [88]. Further study showed that DUX4 directly activates HSATII bidirectional transcription, and that the formation of HSATII dsRNA foci sequesters factors such as ADAR1 and EIF4A3. This may inhibit RNA editing and nonsense-mediated decay (NMD) pathways, respectively, leading to a global dysregulation of transcript quality control in FSHD muscle cells.

Feng et al. (2015) reported that DUX4 promoted the degradation of UPF1, one of the main effectors of NMD [89], through an unknown mechanism [90]. NMD was indeed inhibited, as upon *DUX4* overexpression in immortalized and primary myoblasts there was an observed increase in transcripts with premature translation termination codons prior to splice junctions. Moreover, the *DUX4* transcript is itself a predicted target of NMD. Thus, by inhibiting NMD, DUX4 is able to stabilize its own transcript and positively autoregulate its own expression.

### 3.5. Immune Response Activation

In their siRNA screen, Shadle et al. (2017) saw that knockdown of *RNASEL* and *EIF2AK2* mitigated the toxicity induced by DUX4 in RD-DUX4i cells [54]. Both are effectors of the innate immune response, especially against viral invasion. The presence of dsRNAs activates *RNASEL* and *EIF2AK2* expression, which then proceed to either cleave intruding RNAs or inhibit translation, respectively [91,92]. In a different study, Geng et al. (2012) observed that DUX4 can upregulate the expression of *DEFB103*, producing increased antimicrobial β-defensin 3 peptide levels in the cell [93]. This peptide serves to inhibit the innate immune response, and has roles in activating adaptive immunity [93,94]. Besides its functions in immunity, β-defensin 3 also decreased the expression of genes promoting muscle differentiation, leading to decreased myoblast fusion and MyHC expression [93]. Transcriptomic profiling studies have revealed the involvement of other immune response-associated genes in FSHD and other *DUX4*-expressing systems [25,93]. However, more in-depth study is required to ensure that these genes are genuinely acted upon by DUX4 and not simply induced by the inflammatory environment in FSHD-affected muscle. It would also be interesting to determine if the immune response is involved in the characteristic development of certain FSHD symptoms.

### 3.6. Gene Regulation

Evidence suggests that in addition to being a transcription factor, DUX4 can improve the accessibility of its target gene promoters by inducing local chromatin relaxation. Choi et al. (2016) demonstrated that DUX4 does so via recruitment of p300/CBP, histone acetyltransferases sharing high sequence similarity with each other [95,96,97]. p300 and CBP are known to promote gene expression through several pathways [96,97,98]. DUX4 interacts with both proteins through the last 98 amino acids of the DUX4 C-terminus. Using immortalized myoblasts with doxycycline-inducible *DUX4* expression, the authors found that p300 was recruited to the *ZSCAN4* promoter in *DUX4*-expressing cells but only when the C-terminus was present. This was accompanied by an enrichment of active H3K18ac and H3K27ac marks at the locus, an effect also observed globally across DUX4 target sites. A succeeding study treated similar doxycycline-inducible myoblasts with iP300w, a p300 inhibitor, and reported that it improved cell viability and attenuated DUX4 target gene expression [99]. This effect of DUX4 on global histone acetylation appears to be conserved, as the murine homologue Dux induces a similar phenomenon [99]. It is possible that the involvement of p300/CBP explains how strategies that aim to increase intracellular cyclic adenosine monophosphate (cAMP) levels could inhibit DUX4-mediated toxicity, given that cAMP signalling is a known regulator of p300/CBP activity [84,100,101].

A recent study by Resnick et al. (2019) further showed that DUX4 induces *H3.X* and *H3.Y* expression, as well as causes the increased incorporation of these histone variants into the nucleosomes of its target genes [102]. H3.X and H3.Y are associated with relaxed chromatin regions and have been found to greatly facilitate DUX4 target gene-specific induction and perdurance. Knockdown of *H3.X* and *H3.Y* reduced *DUX4* expression in FSHD patient-derived muscle cells, suggesting a regulatory mechanism for enhancing *DUX4* transcription. Similar mechanisms for DUX4 to promote its own transcription have been observed via the MBD3L protein family, whose expression is likewise induced by DUX4 [103].

These studies paint the picture of DUX4 as being a pioneer transcription factor, which corresponds with its role in very early embryonic development. It is interesting how DUX4 can also create novel promoters in regions containing repetitive elements, such as from mammalian apparent LTR retrotransposons (MaLRs) and endogenous retrovirus elements [93,104]. These were shown to influence the expression of existing genes and/or their antisense counterparts, resulting in transcriptional rewiring. The transcription of these elements themselves is activated by DUX4, similar to what we have seen for HSATII [88].

Alternate potential mechanisms of DUX4-mediated gene regulation have been described. For instance, DUX4 regulates the expression of various miRNAs and lncRNAs [93,105]. However, the functional significance of these dysregulated noncoding RNAs in FSHD is yet to be elucidated. DUX4 may also be regulating gene expression by impacting the architecture of the nucleus. *DUX4* expression disrupts the structure of nuclear bodies, including promyelocytic leukemia (PML) protein bodies and SC35 speckles [106]. Nuclear bodies serve various roles ranging from ensuring genomic integrity to regulating transcription and mRNA splicing [107,108], and thus must be structurally maintained to preserve such functions. Furthermore, nuclear aggregation of proteins such as TDP-43 and FUS [109], as well as dsRNAs [54] may be sequestering critical factors for the transcriptional regulation of other genes, or perhaps initiating novel signalling pathways that could lead to pathological consequences. Future work on these areas will help shed more light on the regulatory landscape of DUX4 at the cellular level.

### 3.7. Other Pathways

Although we have extensively described various pathways participated in by DUX4 in skeletal muscle, it is important to note that this represents but a mere fraction of DUX4-mediated signalling. Advances in transcriptomic and proteomic methods have accelerated our ability to identify global changes in gene expression, and we are extremely fortunate that the FSHD field has been taking advantage of such technologies. Studies have implicated DUX4 in a myriad of pathways in the context of skeletal muscle: RNA metabolism and splicing [110,111,112]; protein translation and homeostasis [109,110,111]; sarcomeric organization [110]; germline and stem cell development [77,93]; extracellular and intracellular transport [111,112]; stress response [111]; cell polarity, adhesion, and migration [112]; and extracellular matrix signalling [112], to name just a few.

With this wealth of information, the field is poised to uncover the mechanisms underlying FSHD pathogenesis as mediated by DUX4. We are still in the early stages of not only understanding all these potentially novel pathways, but also making sense of pathways previously determined to belong to the DUX4 signalling network. For instance, we still do not quite understand how *PITX1* up-regulation contributes to FSHD pathogenesis—and *PITX1* was the first-ever identified direct transcriptional target of DUX4 [47,113]. Due to its role in establishing left-right asymmetry [47,114], PITX1 has been thought to explain the peculiar asymmetric involvement of muscles in FSHD. However, this has not been followed up. An explanation as to why certain gene families are consistently affected by *DUX4* expression (e.g., PRAME, TRIM, MBDL [25]) is also yet to be provided. Revisiting these earlier findings will help clarify the role of previously identified molecular players in FSHD and inform future inquiries into how these may be involved in novel DUX4-mediated signalling pathways.

## 4. Conclusions

FSHD presents the unique case of a muscular disorder caused by the expression of a classically embryonic gene at the wrong place, at the wrong time. Through our survey of the pathways affected by DUX4, we see how the initiation of an embryonic program of gene expression in skeletal muscle leads to overall toxicity and in most cases cell death. Evidence of DUX4 target gene dysregulation can be seen as early as in FSHD-affected fetuses [115,116], suggesting that the FSHD phenotype may be the cumulative result of extensive aberrant signalling across time. This review also helps provide potential explanations as to how *DUX4* expression can translate to the clinical features seen in FSHD patients. However, we are still left with several outstanding questions: What factors determine the age of onset and degree of severity of FSHD symptoms in patients? Why are some mutation carriers asymptomatic? Why are certain muscles affected more than others, and how does this vary from patient to patient? Can genotype–phenotype correlations be made? Whether or not a better understanding of DUX4 signalling will help answer these questions remains to be seen. Integrative analysis approaches will likely be needed considering the inherent complexity of signalling pathways.

It is entirely possible that DUX4 signalling alone may not be sufficient to explain all the aspects of FSHD pathogenesis. Low *FAT1* expression levels have been found to correlate with earlier symptom onset in muscles from patients with infantile FSHD [117]. However, DUX4 and FAT1 do not seem to be influencing each other’s expression. In another study, single-cell RNA sequencing of FSHD patient-derived primary myocytes revealed the presence of a non-DUX4-associated gene signature [118]. Genes located near the D4Z4 repeat array, such as *FRG1* and *FRG2*, have also been inconsistently implicated in FSHD [119,120,121,122]. While both are direct transcriptional targets of DUX4 [120,121], the possibility exists that alterations in the chromatin state of the D4Z4 array could directly influence the transcription of these genes and affect FSHD pathogenesis. In this discussion, we have also mostly focused on the downstream consequences of *DUX4* activation. It is equally possible that events upstream of *DUX4* expression [123] may be involved in helping generate the FSHD phenotype.

Moreover, as a point of caution, results across studies have to be interpreted carefully owing to the fact that the use of different models can lead to different conclusions. Sharma et al. (2013) showed that the top cell death pathways activated by *DUX4* expression depended highly on whether mouse C2C12 or human rhabdomyosarcoma cells were used for the experiment [69]. We have also seen how the dependence of DUX4-mediated apoptosis on p53 was challenged by differences in the *DUX4* overexpression system used [53]. Moving forward, it would be helpful if standardized protocols were developed to study DUX4 and if results could be confirmed in more than one model if applicable. Fortunately, we now have an abundance of *in vitro* and *in vivo* FSHD models available for use [123]. An increased research effort is definitely still required to fully understand the mechanisms of FSHD pathogenesis. With the current pace of knowledge generation in the field, however, it is only a matter of time until these mechanisms are elucidated and eventually used to inform the development of novel therapies for FSHD.

## Abbreviations

FSHD	facioscapulohumeral muscular dystrophy
DUX4	double homeobox protein 4
ORF	open reading frame
AAV	adeno-associated virus
ROS	reactive oxygen species
RTK	receptor tyrosine kinase
NMD	nonsense-mediated decay
NADPH	reduced nicotinamide adenine dinucleotide phosphate
cAMP	cyclic adenosine monophosphate
MaLR	mammalian apparent LTR retrotransposon
PML	promyelocytic leukemia
